# The Antimicrobial Potency of Mesoporous Silica Nanoparticles Loaded with *Melissa officinalis* Extract

**DOI:** 10.3390/pharmaceutics16040525

**Published:** 2024-04-10

**Authors:** Gabriela Petrișor, Ludmila Motelica, Roxana Doina Trușcǎ, Andreea-Luiza Mȋrț, Gabriel Vasilievici, Justinian-Andrei Tomescu, Cristina Manea, Andreea Ștefania Dumbravǎ, Viorica Maria Corbu, Irina Gheorghe-Barbu, Denisa Ficai, Ovidiu-Cristian Oprea, Bogdan-Ștefan Vasile, Anton Ficai, Anca Daniela Raiciu

**Affiliations:** 1 Science and Engineering of Oxide Materials and Nanomaterials, Faculty of Chemical Engineering and Biotechnologies, National University of Science and Technology POLITEHNICA Bucharest, Gh. Polizu 1-7, 011061 Bucharest, Romania; gabriela.petrisor06@yahoo.com (G.P.); ludmila.motelica@upb.ro (L.M.); roxana_doina.trusca@upb.ro (R.D.T.); luiza.mirt@yahoo.com (A.-L.M.); bogdan.vasile@upb.ro (B.-Ș.V.); anton.ficai@upb.ro (A.F.); 2 National Center for Scientific Research for Food Safety, National University of Science and Technology POLITEHNICA Bucharest, Splaiul Independentei 313, 060042 Bucharest, Romania; denisa.ficai@upb.ro; 3 National Center for Micro and Nanomaterials, National University of Science and Technology POLITEHNICA Bucharest, Splaiul Independentei 313, 060042 Bucharest, Romania; 4 Academy of Romanian Scientists, Ilfov Street 3, 050044 Bucharest, Romania; 5 National Institute for Research & Development in Chemistry and Petrochemistry–ICECHIM, Spl. Independentei 202, 060021 Bucharest, Romania; gvasilievici@icechim.ro; 6 S.C. Hofigal Export-Import S.A., Intrarea Serelor 2, 042124 Bucharest, Romania; tomescu.justinian@gmail.com (J.-A.T.); directorgeneral@hofigal.eu (C.M.); daniela_raiciu@yahoo.com (A.D.R.); 7 Faculty of Biology, University of Bucharest, 1-3 Aleea Portocalelor, 060101 Bucharest, Romania; andreeadum29@gmail.com (A.Ș.D.); viorica.corbu@yahoo.com (V.M.C.); iryna_84@yahoo.com (I.G.-B.); 8 Department of Inorganic Chemistry, Physical Chemistry and Electrochemistry, Faculty of Chemical Engineering and Biotechnologies, National University of Science and Technology POLITEHNICA Bucharest, Gh. Polizu 1-7, 011061 Bucharest, Romania; 9 Department of Pharmacognosy Phytochem Phytoterapy, Faculty of Pharmacy, Titu Maiorescu University, Gh. Sincai 16, 040405 Bucharest, Romania

**Keywords:** Melissa officinalis, MCM-41, MCM-48, APTES, antimicrobial activity, hydroalcoholic extract, methicillin-resistant *S. aureus*, MRSA

## Abstract

*Melissa officinalis* is an important medicinal plant that is used and studied intensively due to its numerous pharmacological effects. This plant has numerous active compounds with biomedical potential; some are volatile, while others are sensitive to heat or oxygen. Therefore, to increase stability and prolong biological activities, the natural extract can be loaded into various nanostructured systems. In this study, different loading systems were obtained from mesoporous silica, like Mobile Composition of Matter family (MCM) with a hexagonal (MCM-41) or cubic (MCM-48) pore structure, simple or functionalized with amino groups (using 3-aminopropyl) such as triethoxysilane (APTES). Thus, the four materials were characterized from morphological and structural points of view by scanning electron microscopy, a BET analysis with adsorption–desorption isotherms, Fourier-transform infrared spectroscopy (FTIR) and a thermogravimetric analysis coupled with differential scanning calorimetry. Natural extract from *Melissa officinalis* was concentrated and analyzed by High-Performance Liquid Chromatography to identify the polyphenolic compounds. The obtained materials were tested against Gram-negative bacteria and yeasts and against both reference strains and clinical strains belonging to Gram-positive bacteria that were previously isolated from intra-hospital infections. The highest antimicrobial efficiency was found against Gram-positive and fungal strains. Good activity was also recorded against methicillin-resistant *S. aureus*, the *Melissa officinalis* extract inhibiting the production of various virulence factors.

## 1. Introduction

**Scheme 1:**
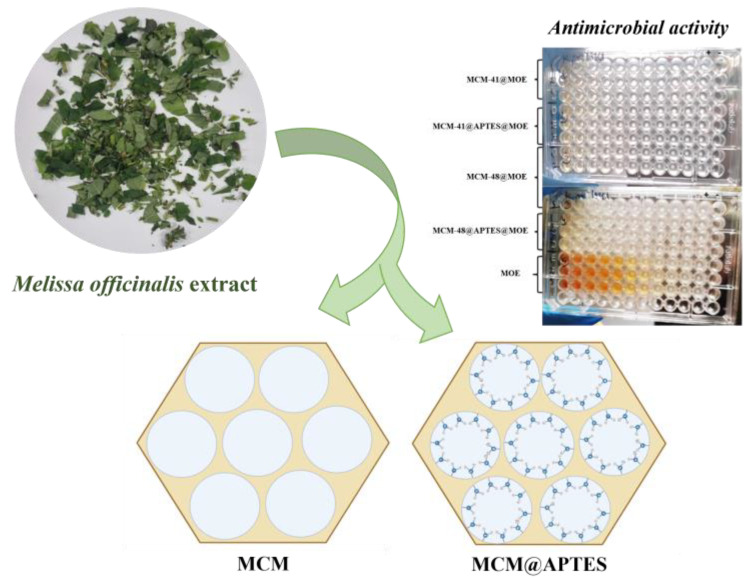
Synthesis of systems from mesoporous silica loaded with *Melissa officinalis* extract and their antimicrobial activity.

Medicinal plants are of particular importance in current studies because the active substances in their compositions display numerous biological activities [1]. *Melissa officinalis* or lemon balm is a medicinal plant from the Lamiaceae family [2] and is recognized for its pharmacological effects, such as antimicrobial [3,4], antibacterial [5], cytotoxic [6,7,8], antidepressant [9,10], neuroprotective [11], cardioprotective [12], anti-inflammatory effects [13] and so on. *Melissa officinalis* has strong antioxidant activity [7,14] due to the presence of polyphenolic compounds found in high concentrations. The polyphenol found as the most abundant compound in the composition of *Melissa officinalis* is rosmarinic acid, followed by caffeic acid, gentisic acid, chlorogenic acid, caftaric acid, ferulic acid and p-coumaric acid [15]. Current research aims to load mesoporous silica systems with natural extracts from *M. officinalis*. The materials used have the role of carrier and can have different structures so that the extract or active substance can be easily loaded. The carriers presented in literature are found in the forms of emulsions, gels, vesicles, nanospheres, polymer particles, capsules, phytosomes, etc. [16,17,18,19]. Mesoporous silica nanoparticles have been found to have useful properties to be used as carriers, such as good compatibility, high specific surface area, variable pore sizes and controllable pore volumes [20,21,22,23]. We chose to use two types of mesoporous silica as carriers, MCM-41 and MCM-48, which belong to the MCM (Mobile Composition of Matter) family [24]. The major difference between the two materials is that they have different pore structures; it is hexagonal in MCM-41 and cubic in MCM-48 [25]. Carriers can be functionalized with different substances for good optimization of the system, so that the active substance is loaded and released depending on its utility [26,27,28], even with an amplified efficiency. It was reported that functionalized mesoporous silica nanoparticles (MSNs) with cefepime and meropenem exhibit higher antimicrobial activity against *Acinetobacter baumannii* strains than free cephalosporin or carbapenem antibiotics [29]. Similarly, higher antimicrobial activity against *Staphylococcus aureus* isolated from an intracellular infection was reported for functionalized MSNs with rifampicin [30], and excellent antimicrobial activity was found for rutin-silver-decorated MSNs against Gram-negative bacteria (*Escherichia coli*), Gram-positive bacteria (*S. aureus*) and different *Candida albicans* and *Candida* non-*albicans* species [31]. Thus, (3-aminopropyl) triethoxysilane (APTES) was used for the functionalization of mesoporous silica nanoparticles with amino groups (-NH_2_), and both simple and functionalized mesoporous silica systems were loaded with *M. officinalis* extract (MOE), as shown in Scheme 1.

In this context, our aim was to demonstrate the effectiveness of *Melissa officinalis* extract (MOE) loaded on MSNs (Scheme 1) as an alternative against both reference and clinical strains belonging to Gram-positive strains that were previously obtained from intra-hospital infections, as well as against Gram-negative bacteria and yeast. As MSN support might influence the biological activity of MOE, we tested a series of MSNs belonging to the Mobile Composition of Matter family (MCM) with hexagonal (MCM-41) or cubic (MCM-48) pore structures, both simple or functionalized with amino groups from APTES.

## 2. Materials and Methods

### 2.1. Materials

Tetraethyl orthosilicate (TEOS), cetyltrimethylammonium bromide (CTAB), ammonia (NH_3_) and absolute ethanol from Merck (Darmstadt, Germany) were used to obtain the MCM-41 type mesoporous material. Functionalization of mesoporous silica was carried out with (3-aminopropyl) triethoxysilane (APTES) purchased from Sigma Aldrich (St. Louis, MO, USA).

All materials were prepared in distilled water (dw), and all substances were used without any additional purification.

For the chromatographic analysis, chromatographic purity reagents were used; Folin–Ciocalteu reagent, orthophosphoric acid (H_3_PO_4_) and acetonitrile were purchased from VWR chemicals (Radnor, PA, USA). Reference standards: rosmarinic acid was obtained from Cayman Chemical Company (Ann Arbor, MI, USA), and caftaric, chlorogenic, caffeic and chicoric acids were obtained from PhytoLab GmbH & Co (Vestenbergsgreuth, Germany).

Aerial parts of *Melissa officinalis* from S.C. Hofigal Export-Import S.A. (Bucharest, Romania) culture were used to obtain the natural extract, namely *Melissa officinalis* extract (MOE).

### 2.2. Equipment

The obtained mesoporous materials were structurally characterized by Fourier-transform infrared spectroscopy (FTIR) with a Nicolet iS 50 (Thermo Fisher Scientific, Waltham, MA, USA) device equipped with an attenuated total reflectance module (ATR). Using the N_2_ adsorption isotherms, the Brunauer–Emmett–Teller (BET) analysis was effectuated with the help of a NOVA 2200e Gas Sorption Analyzer (Quantachrome, Boynton Beach, FL, USA) in the relative pressure interval p/p0 = 0.005–1.0 and at 77.35 K. The materials were outgassed under a vacuum at 110 °C for 4 h just before the measurements began. A QUANTA INSPECT F electron microscope (FEI Company, Eindhoven, The Netherlands) was used to determine the surface morphology of mesoporous silica. The device is equipped with a field emission gun and an energy-dispersive (EDS) detector. Prior examination samples were coated with a thin layer of silver for better conductivity. Visualization of particles’ sizes and mesopores’ orders was carried out with a Tecnai G2F30 S-TWIN high-resolution transmission electron microscope (TEM) from FEI (FEI Company, Eindhoven, the Netherlands). A Netzsch 449C STA Jupiter thermo-balance (Selb, Germany) was used for thermogravimetric analyses up to 900 °C. The open crucible was heated with a rate of 10 °C/min in a continuous air flux (50 mL/min).

### 2.3. Obtaining and Characterizing the Melissa officinalis Extract

Aerial parts of *Melissa officinalis* from S.C. Hofigal Export-Import S.A., Bucharest, Romania, own culture, were used to obtain the *Melissa officinalis* extract (MOE) as follows: The aerial parts of *M. officinalis*, after harvest, were dried at 40 °C until 8% humidity was reached, and then they were crushed using the Retsch laboratory mill with a knife (Grindomix GM 200 model); the work schedule was 30 s at 10,000 RPM (rotations per minute). Then, 100 g of plant material powder was weighed on an analytical balance and macerated in 500 mL of solvent (50% ethanol in water, *v*/*v*) in a ratio of 1:5 (plant/solvent, *w*/*v*) with periodic stirring. After 5 days, it was filtered, and the obtained liquid extract was concentrated in a stream of nitrogen until the volume of 85 mL of concentrated extract was obtained. This was further considered the *Melissa officinalis* extract (MOE) for this research.

After obtaining the concentrated extract of *M. officinalis*, it was subjected to separation analysis to identify the polyphenolic compounds. An analysis was performed on a C18 Zorbax SB reversed-phase column (Agilent, Santa Clara, CA, USA) (150 × 4.6 mm) using a Thermo Fisher Scientific Vanquish™ Core High-Performance Liquid Chromatography (HPLC), (Waltham, MA, USA) system equipped with a Vanquish™ Core Dual C pump (VC- P32-A-01), Vanquish Split Autosampler (VC-A12-A-02), Vanquish™ Column Compartment (VC-C10-A-03) and Vanquish™ Diode Detector (VC-D11-A-01). A sample (2 µL) was injected and then eluted using mobile phase A consisting of 0.1% H_3_PO_4_ and mobile phase B, acetonitrile, using the following gradient: 10-17% B, 0–4 min; 17–22% B, 4–5 min; 22% B, 5–7 min; 22–40% B, 7–8 min; 40–10% B, 8–9 min; 10% B, 9–11 min. The identification of compounds was performed by comparison with the retention time of standard phenolic acids.

### 2.4. Preparation of Mesoporous Materials and Adsorption of MOE

The synthesis of MCM-41 and MCM-48 mesoporous silica and their functionalization were carried out under the same conditions described previously [32,33,34]. MCM-41 and MCM-48 were synthesized using the soft template method in a basic medium starting from the silica precursor, TEOS, and the template agent, CTAB, which auto-assembles into micelles that generate hexagonal or cubic pores as a silica network is formed by hydrolysis and condensation reactions. By calcination, the CTAB template was removed, and the mesoporous materials were obtained. Briefly 0.5 g of CTAB was dissolved in 96 mL of distilled water and sonicated until the solution became clear. Subsequently, 34 mL of ethanol and 10 mL of an ammonia solution were added, and stirring was continued until the solution became homogeneous. After mixing, 2 mL of TEOS was added and stirred for an additional three hours at the same speed of rotation, and a translucent precipitate was obtained. The obtained precipitate was filtered and washed with distilled water and ethanol. The last step of the synthesis consisted of purification/washing with ethanol (20 mL) and water (three times with 20 mL) and drying for 12 h at 100 °C. The final product was annealed for nine hours at 550 °C according to the following program: 0–300 °C at 80 °C/min and 300–550 °C at 20 °C/min [33]. This mesoporous material was labeled MCM-41. By using 2 g of CTAB, the MCM-48 mesoporous structure was obtained.

The functionalization of mesoporous silica was achieved by a coupling reaction of MCM-41 and MCM-48 with the coupling agent, APTES. Briefly, one gram of each mesoporous material (MCM-41 and MCM-48) was dried in a vacuum drying oven at a temperature of 23 °C for 3 h. After drying, 100 mL of ethanol was added, and the suspensions were sonicated for one hour. An amount of 125 μL of APTES was added to each suspension, left to reflux for 24 h and sonicated for one hour. The obtained suspensions were filtered and left to dry [32].

The pores of the mesoporous materials MCM-41, MCM-48, MCM-41@APTES and MCM-48@APTES allow for the loading of bioactive compounds from the hydroalcoholic extract of *M. officinalis* by their adsorption under vacuum. The experiment began with adding one gram of non-functionalized mesoporous material (MCM-41/MCM-48) to a glass vessel and maintaining it for 30 min under a vacuum. For more efficient loading, 5 mL of MOE was added in two steps so that the compounds did not settle on the surface of the material. The adsorption of MOE in the functionalized mesoporous materials (MCM-41@APTES and MCM-48@APTES) was carried out under the same conditions, and it is worth mentioning that the adsorption of the extract took longer. After loading the materials, they were kept in an oven at a temperature of 60 °C, and thus, we finally obtained the materials mentioned in Table 1.

### 2.5. Determination of Total Polyphenol Content (TPC)

The concentrated extract of *M. officinalis* was evaluated by the Folin–Ciocâlteu method [35] to obtain the Total Polyphenol Content (TPC). The extract was mixed with 5 mL of Folin–Ciocâlteu 10% phenol reagent and allowed to stand for 3 to 5 min at room temperature. After incubation, 4 mL of 7.5% Na_2_CO_3_ was added, and the reaction mixture was mixed well and allowed to stand for 1 h at room temperature. Absorbance was measured with a Jasco Spectrophotometer UV-VIS V-530 (JASCO Inc., Easton, PA, USA), and the total polyphenolic content was calculated using the standard calibration curve of caffeic acid and chlorogenic acid.

### 2.6. Qualitative Testing of Antimicrobial Activity against Reference Microbial Strains

The antimicrobial activity of the MOE-loaded MSNs was determined against Gram-positive bacterial strains, namely *Staphylococcus aureus* ATCC 6538 and *Enterococcus faecium* ATCC 29212; Gram-negative bacterial strains, namely *Escherichia coli* ATCC 13846, *E. coli* ATCC 8732, *Enterobacter aerogenes* ATCC 13048, *Pseudomonas aeruginosa* ATCC 28792 and *Klebsiella pneumoniae* ATCC 13368; and the yeast strain *Candida albicans* ATCC 10231. All reference strains were purchased from the American Type Culture Collection (ATCC, Washington, DC, USA).

The strains were maintained on a liquid medium supplemented with 20% glycerol Luria–Bertani broth from Sigma Aldrich (Redox Lab Supplies, Bucharest, Romania) in the case of bacteria, and on Yeast Peptone Glucose Broth (YPGB) medium (0.5% yeast extract; 1% peptone; 0.2% glucose) in the case of *Candida* strains at −70 °C (Revco LegaciTM Refrigeration System ((Thermo Fisher Scientific, Waltham, MA, USA)), respectively. Before running the experiments, each strain was subcultured on the appropriate culture medium and cultivated for 24 h at 37 °C.

For the qualitative testing of antimicrobial activity, an adapted disc diffusion method was used [36,37]. From fresh microbial cultures (24 h, 37 °C), 0.5 McFarland (1.5 × 10^8^ CFU/mL) suspensions were prepared in 0.85% NaCl solution. The microbial suspensions were inoculated on specific culture media (Mueller–Hinton Agar (MHA) (Oxoid) for bacteria and Mueller–Hinton supplemented with 2% glucose and 0.5 µg/mL of methylene blue (MHG) for *C. albicans* ATCC 10231. From each sample (10 mg/mL), respectively, the undiluted *M. officinalis* extract and the solvent used to obtain the plant extract (v:v water/ethanol mixture) were spotted (10 µL spot), and the plates were incubated for 24 h at 37 °C. Antimicrobial activity was quantified by measuring the diameters of the growth inhibition zones and expressed as arbitrary units: arbitrary unit 1—diameter <10 mm; arbitrary unit 2—diameter > 10mm.

The determination of the minimum inhibitory concentration (MIC) was performed using binary micro-dilutions in 96-well plates in MHB medium for bacteria and RPMI—Roswell Park Memorial Institute 1640—supplemented with L-glutamine and 25 mM of Hepes in the case of the *C. albicans* ATCC 10231 strain (final volume of 100 µL) [38]. Each well was inoculated with 10% 0.5 McFarland microbial suspension and subsequently incubated for 24 h at 37 °C. At the end of the incubation period, microbial growth was quantified spectrophotometrically by determining the absorbance at 600 nm using the BIOTEK SYNERGY-HTX ELISA multireader (Agilent, Santa Clara, CA, USA). The MIC value was established as the concentration value at which the complete inhibition of microbial growth was observed (OD_600nm_ ≤ 0.02 after blank subtraction). All tests were conducted in triplicate, and values were presented as mean ± standard deviation. Similarly, the antimicrobial activity was tested against a total of seven methicillin-resistant *S. aureus* (MRSA) strains that were previously isolated from intra-hospital infections and investigated regarding their antimicrobial susceptibilities using the VITEK 2 system (resistance to oxacillin, erythromycin and clindamycin) and preserved in the collection of microorganisms in the Center for Research, Training and Consultation in Genetics, Microbiology and Biotechnology, Faculty of Biology, University of Bucharest.

### 2.7. The Influence of MOE-Loaded MSNs on the Adherence Ability of Microbial Strains to an Inert Substrate

The influence of MOE-loaded MSNs on the ability of microbial strains to adhere to an inert substrate was determined using the crystal violet microtitration method. Thus, the microbial strains were cultured in the presence of subinhibitory concentrations of materials (MIC/2–half of MIC value; MIC/4–quarter of MIC value) according to the previously described protocol for the quantitative evaluation of antimicrobial activity. At the end of the incubation period, cells adhered to the inert substrate were fixed with 99% methanol, stained with 1% crystal violet solution and resuspended in 33% acetic acid solution. The percentage of microbial adherence inhibition (MAI%) was established according to the following relationship: MAI% = (As − Ablank) × 100/(Ac − Ablank). As is the value of absorbance at 490 nm of samples treated with a sub-inhibitory concentration of materials, and Ac is the value of absorbance at 490 nm of the control (microbial strain not treated with materials) [39].

### 2.8. The Influence of MOE-Loaded MSNs on the Ability to Secrete Soluble Virulence Factors

*S. aureus* strains cultivated in the presence of MOE-loaded MSNs (MIC/2) were evaluated for their ability to secrete soluble virulence factors, such as hemolysins, lecithinase, amylase, lipase, caseinase, gelatinase and esculin hydrolysis, as previously described [40]. The influence on the ability to secrete soluble virulence factors was quantified using the following relationship: % inhibition = (D2 − C2) × 100/(D1 − C1) × 100. C1—the diameter of the culture spot untreated with the control of the MOE-loaded MSNs; D1—the diameter of the clear/precipitated zone around the culture spot untreated with the control of the MOE-loaded MSNs; C2—the diameter of the culture spot treated with a sample of MIC/2 MOE-loaded MSNs; D2—the diameter of the clear/precipitated zone around the culture spot treated with a sample of MIC/2 MOE-loaded MSNs.

### 2.9. Statistical Analysis

The obtained data were expressed as means ± SD. Statistical analysis was performed using GraphPad Prism v9.3.0. Data were analyzed using two-way ANOVA for the adherence and inhibition of virulence factors. Correction of multiple comparisons regarding the inhibitory effect of mesoporous material solutions on adhesion to inert substrate was performed using Dunnett’s test, comparing control values versus MIC/2 and MIC/4 values. Correction for multiple comparisons to determine the effect of mesoporous materials on the production of soluble virulence factors was performed using the Dunn-Šidák method. The level of statistical significance was set at *p* < 0.05.

## 3. Results and Discussion

### 3.1. Characterization of M. officinalis Plant Extract

**Figure 1:**
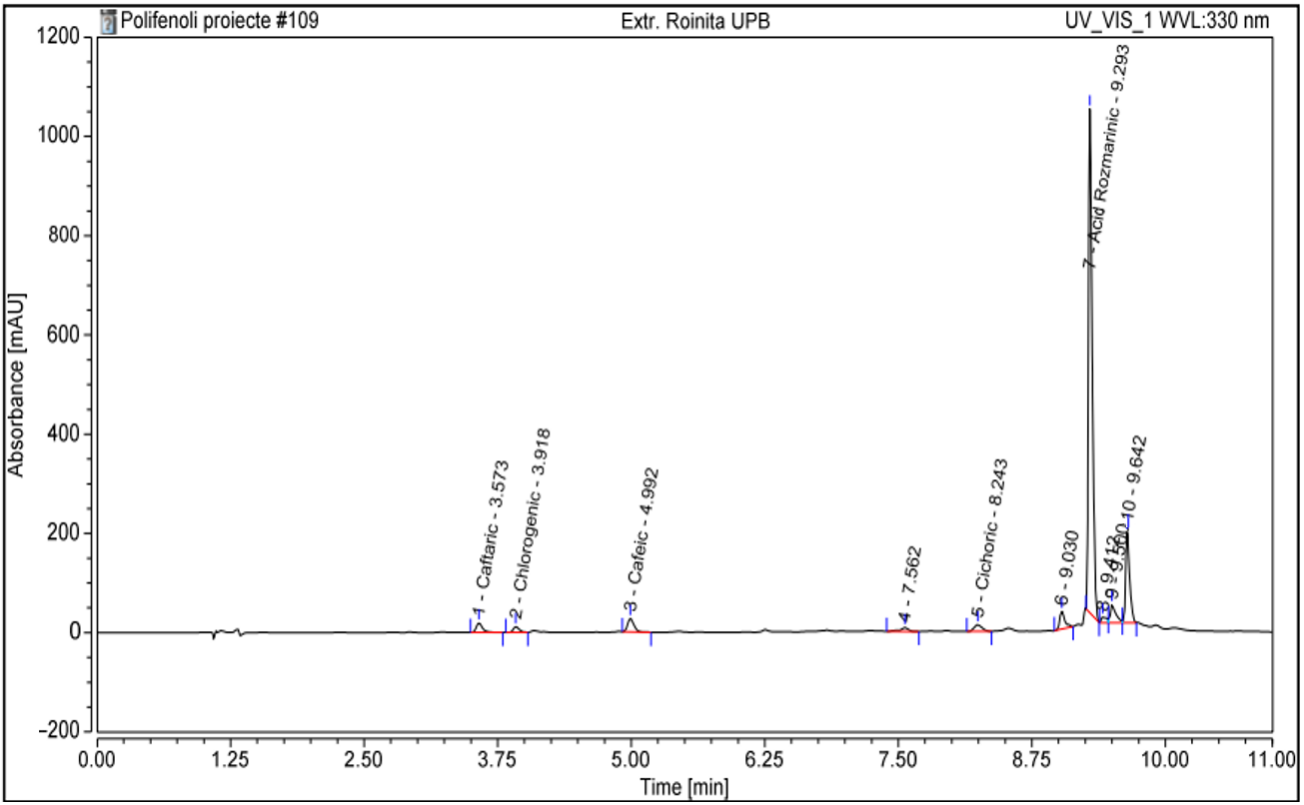
HPLC chromatogram for separation of polyphenolic compounds from MOE.

*M. officinalis* extract has a composition that is rich in polyphenols and flavonoids that depends on several factors, such as the type of culture, locality, temperature, soil and so on. Through the HPLC analysis of the concentrated extract of *M. officinalis*, five components were identified (Figure 1, Table 2), among which rosmarinic acid had the highest concentration of 1286 mg per 100 g of extract. Figure 1 shows the separation of polyphenolic compounds and the peak related to rosmarinic acid with the highest absorbance identified. Regarding the concentration of rosmarinic acid, the concentrations of the other compounds are much smaller [15].

Rosmarinic acid can be found in high concentrations in the *Lamiaceae* family [41] and demonstrate strong antioxidant [42] and antimicrobial activities [43].

Through the Folin–Ciocalteu method, high values were obtained for the total polyphenolic content, with 3.14 g of caffeic acid equivalent to 100 g and 6.33 g of chlorogenic acid equivalent to 100 g (Table 3).

### 3.2. Characterization of MSNs Loaded with M. officinalis Extract

**Figure 2:**
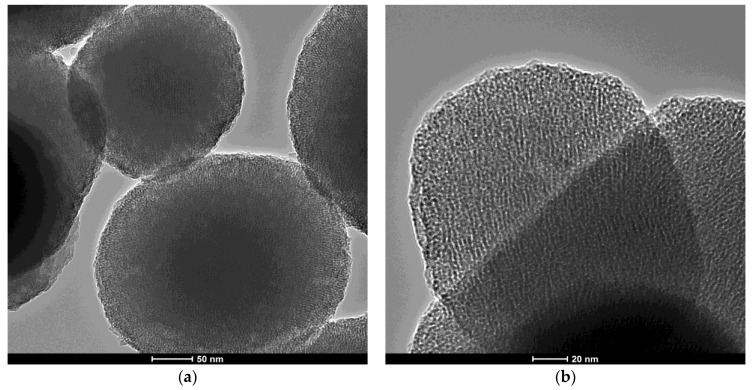
TEM micrographs for MCM-41 (**a**) and MCM-48 (**b**) particles.

The transmission electron micrographs of MCM-41 and MCM-48 are presented in Figure 2. The mesoporous nature of the silica particles can be clearly seen for both the MCM-41 and MCM-48 types, but the measurement of the pores is not possible at this magnification. The overall morphology of the particles is spherical or quasi-spherical.

The scanning electron micrographs for MCM-41 and MCM-48 (Figure S1) indicate the spherical morphology of the silica particles, with variable sizes in the range of 200–400 nm, with a light agglomeration tendency.

The mesoporous silica particles loaded with *M. officinalis* extract (1 g of MSN was loaded with 5 mL of MOE) were characterized using scanning electron microscopy, a BET analysis with adsorption–desorption isotherms, Fourier-transform infrared spectroscopy (FTIR) and thermogravimetric analysis (TG) with differential scanning calorimetry (DSC) values.

**Figure 3:**
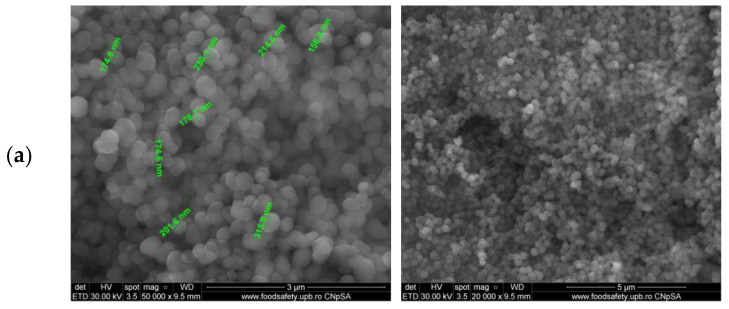

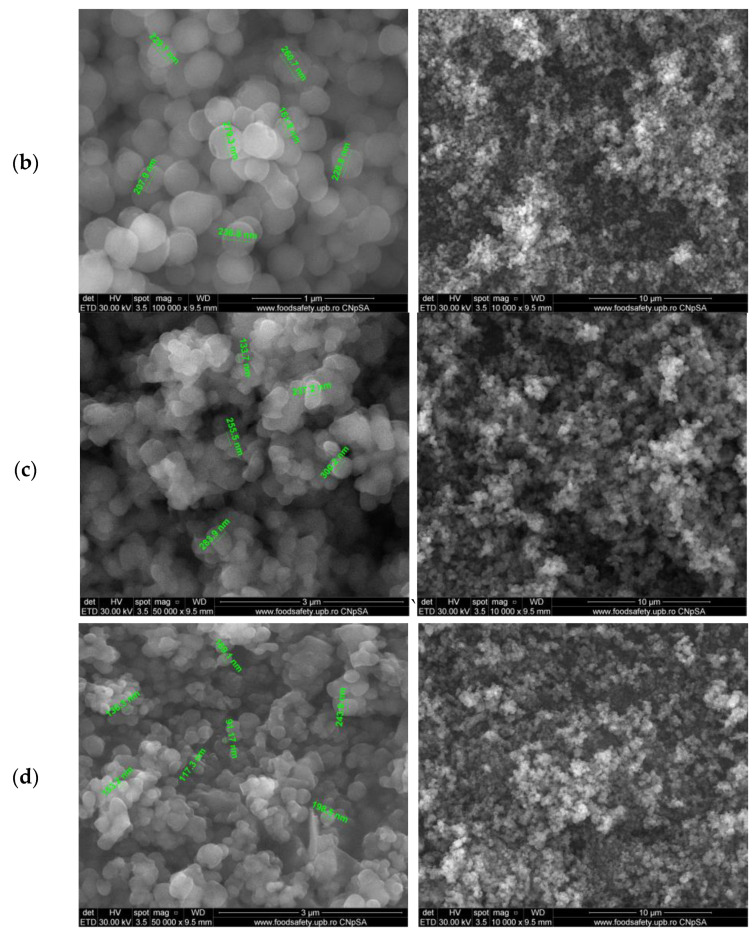
SEM micrographs of (**a**) MCM-41@MOE, (**b**) MCM-41@APTES@MOE, (**c**) MCM-48@MOE and (**d**) MCM-48@APTES@MOE.

The scanning electron microscopy images highlight the structure and morphology of the porous materials, and differences between extract-loaded mesoporous materials can be observed. In Figure 3a,b, we can see MOE-loaded MCM-41 samples consisting of highly homogeneous 150–350 nm particles with a spherical shape. Compared to MCM-41 [32], the SEM images of MCM-41@MOE and MCM-41@APTES@MOE show some heterogeneous areas, which can be attributed to the presence of MOE compounds on the surface. In the SEM images recorded for the mesoporous materials based on MCM-48 in Figure 3c,d, agglomerated silica particles with variable sizes between 100 and 300 nm can be observed. Compared to the SEM images at high magnification, which have MCM-41 as support, more particle agglomerations are observed in the case of materials with MCM-48 support due to the quasi-spherical silica particles. To highlight that the particles were loaded with MOE, the previously reported SEM images of the MCM-41 and MCM-48 samples [32] are more translucent compared to those of the MOE-loaded materials (Figure 3).

The structural characteristics of the MSNs loaded with MOE are presented in Table 4, and when comparing them with the initial surface values of MCM-41 (1365 m^2^/g) and MCM-48 (1582 m^2^/g), a significant decrease in the surface of the materials can be observed [32]. The BET surface area and pore volume decreased a lot, which means that the natural compounds from the extract were loaded inside the pores.

**Figure 4:**
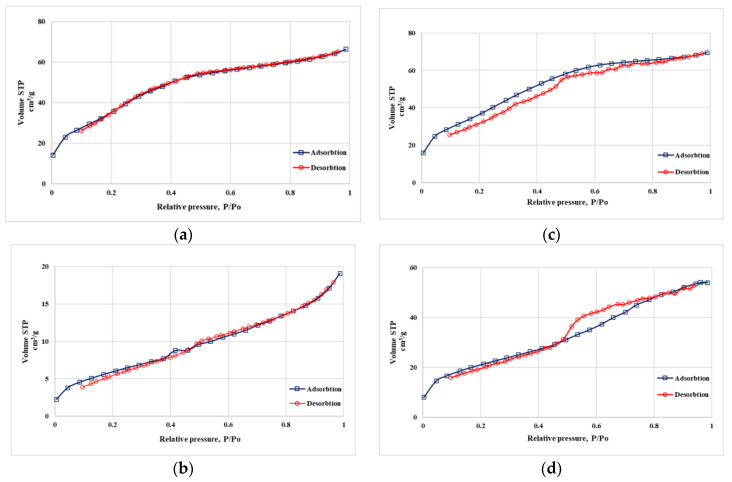
Nitrogen adsorption–desorption isotherms of (**a**) MCM-41@MOE, (**b**) MCM-41@APTES@MOE, (**c**) MCM-48@MOE and (**d**) MCM-48@APTES@MOE.

Referring to the surface area of the unloaded materials from our previous report [32], and considering that the four materials were loaded with the same amount of hydroalcoholic extract, it can be seen that the surface area decreased by 9.48 times for MCM-41@MOE and by 11 times for MCM-48@MOE. The major difference is that for the functionalized materials, the surface area has much lower values compared to the unloaded materials, specifically 45.2 times less for MCM-41@APTES@MOE and 20.42 times less for MCM-48@APTES@MOE. After the functionalization process, the surface area and volume of the pores decrease, which is normal as APTES is creating a new layer on the surface and inside and outside of the pores. Nevertheless, functionalization with amino groups leads to a higher filling percent of the pores with MOE, indicating a better adsorption efficiency. The nitrogen adsorption–desorption isotherms (Figure 4) are well correlated with the values obtained for the surface area and pore volume.

**Figure 5:**
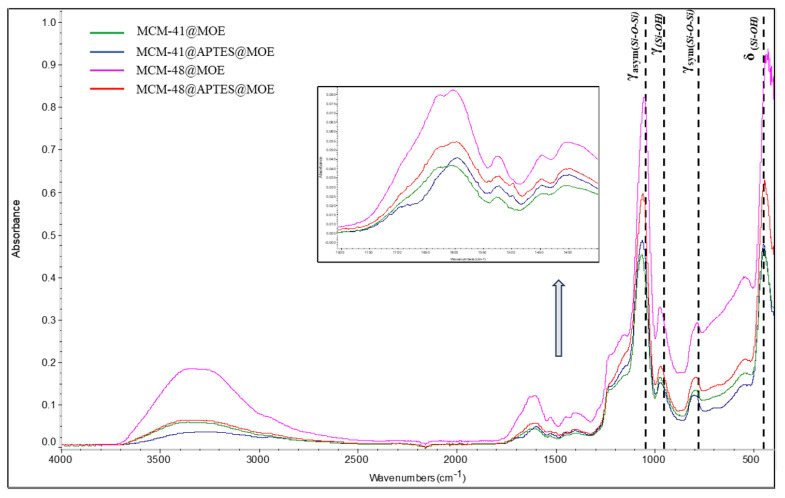
FTIR spectra and band assignments for samples loaded with MOE.

The FTIR spectra of the mesoporous silica loaded with *M. officinalis* extract (Figure 5) highlight the presence of the silica support but also of the natural compounds in the extract.

The silica support is recognized by the presence of absorption bands at ~1213 cm^−1^ and ~1050 cm^−1^ that can be attributed to the stretching vibrations of the asymmetric Si-O-Si and the band associated with the silanol group at ~970 cm^−1^ (Figure S2). The absorption band at ~785 cm^−1^ is generated by stretching vibrations of the symmetric Si-O-Si, and the band at ~437 cm^−1^ is generated by the Si-O-Si moiety’s deformation vibrations [5,44]. After functionalization with APTS, the FTIR spectra for MCM-41@APTS and MCM-48@APTS present small bands at 2980 cm^−1^, which are assigned to C-H vibration (Figure S2).

The FTIR spectra for MOE (Figure S2) present a broad band at 3330 cm^−1^, due to O-H bond vibration, from phenolic groups and solvent. The peaks in the 2800–3000 cm^−1^ region are due to asymmetric and symmetric C-H vibrations, while the peak at 1629 cm^−1^ can be assigned to C-O stretching. By loading the mesoporous silica support with the hydroalcoholic extract of *M. officinalis*, the absorption bands in the ~3321 cm^−1^ area which are characteristic of the stretching vibrations of the O-H bonds corresponding to phenolic groups can be observed, and at ~1593 cm^−1^, it is attributed to the fingerprint region of C-O stretching [45]. Due to the higher molar absorptivity of silanol groups, it is not possible to identify the absorption bands corresponding to symmetric deformation groups such as -CH_3_ or to C-O stretching vibrations. Also, in the case of MCM-41@APTES@MOE and MCM-48@APTES@MOE materials, the absorption bands of the N-H bond associated with NH_2_ that is normally present around 714 cm^−1^ cannot be identified [46].

Successful functionalization with APTES of the MCM-41 and MCM-48 samples is indicated by the different TG/DSC curve shapes (Figure S3). The pristine MCM-41 and MCM-48 samples present a continuous, slow mass loss up to 900 °C. This is generated by the loss of adsorbed water molecules under 200 °C and by the condensation of Si-OH moieties, followed by silica densification [32]. The residual mass at 900 °C is 95.53% for MCM-41 and 97.15% for MCM-48. The APTES-functionalized samples (Figure S3) exhibit a higher mass loss, especially after 200 °C, when the organic part is degraded by oxidation processes (strong exothermic effects can be noticed around 310 °C).

**Figure 6:**
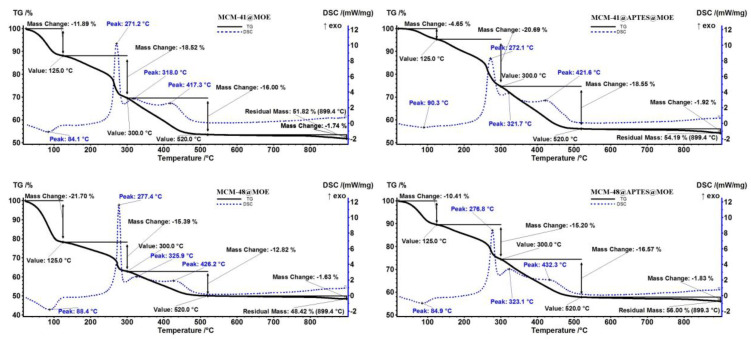
The TG-DSC curves for the MOE-loaded silica samples.

**Figure 7:**
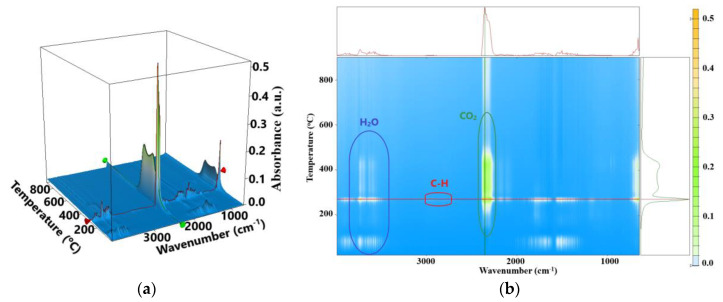
The 3D FTIR diagram for evolved gases from the thermal analysis of MCM-41@MOE (**a**) and its 2D projection in temperature/wavenumber space (**b**); the red line corresponds to the FTIR spectrum at 271 °C, and the green line corresponds to the CO_2_ elimination trace (at wavenumber 2355 cm^−1^).

The thermal analysis for all four samples loaded with MOE follows a similar path as the principal component to be degraded is the *M. officinalis* extract. For the MCM-41@MOE sample, a mass loss of 11.89% up to 125 °C can be observed, and the process is accompanied by an endothermic process with minimum at 84.1 °C on the DSC curve (Figure 6). This process can be assigned to the elimination of residual solvent molecules and volatile components from MOE. In the temperature interval of 125–300 °C, the sample loses 18.52% of its initial mass, with the associated DSC effect being exothermic, strong and sharp, with a maximum at 271.2 °C. This process can be attributed to the partial oxidation of the organic substances loaded onto the mesoporous silica, as indicated by the FTIR of evolved gases at this temperature (Figure 7). After 300 °C, the residual carbonaceous mass is burned away in two separate events, as indicated by the exothermic effects from the DSC curve with maxima at 318.0 and 417.3 °C. The separate events are also visible on the CO_2_ trace (wavenumber 2355 cm^−1^) as individual peaks (Figure 7). The residual mass of 51.82% permits the estimation of the loaded MOE quantity at 46.45%.

The principal data from thermal analysis for all samples are presented in Table 5. It can be seen that simple MCM-41 and MCM-48 samples have higher loading capacities than the corresponding MCM-41@APTES and MCM-48@APTES samples, as indicated by the BET values.

### 3.3. Characterization of MSNs Loaded with M. officinalis Extract

**Figure 8:**
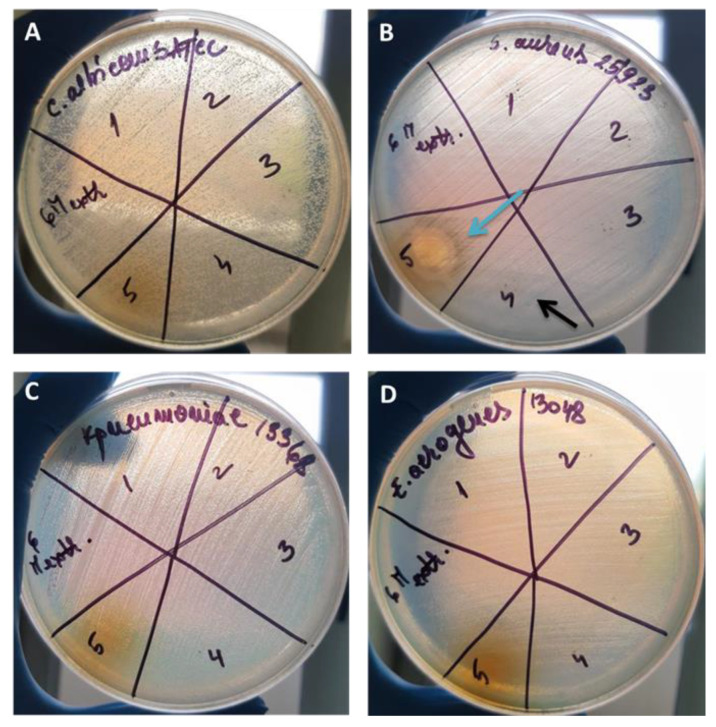
Qualitative evaluation of antimicrobial activity against (**A**) *C. albicans* ATCC 10231; (**B**) *S. aureus* ATCC 25923; (**C**) *K. pneumoniae* ATCC 13368 and (**D**) *E. aerogenes* ATCC 13048. Blue arrow—zone of inhibition, arbitrary unit 2; black arrow—sample that did not inhibit growth of microbial strain; 1—MCM-41@MOE; 2—MCM-41@APTES@MOE; 3—MCM-48@MOE; 4—MCM-48@APTES@MOE; 5—MOE; 6—solvent.

Following the qualitative testing of the antimicrobial activity against Gram-positive and Gram-negative bacterial strains and against *C. albicans* ATCC 10231, it was found that, except for the *M. officinalis* MOE, the other samples do not determine the appearance of inhibition zones/halo; thus, the samples do not present a strong inhibitory effect. In the case of the plant extract, except for *S. aureus* strain ATCC 25923, where the zone of inhibition is clear and has a diameter corresponding to arbitrary unit 2 (Figure 8B, blue arrow), the diameters of the zones of inhibition fall within arbitrary unit 1. Also, isolated colonies with heteroresistance are observed inside the inhibition zone. Taking into account the fact that the plant extract was obtained using a water/ethanol *v:v* mixture as a solvent, in the present study, its inhibitory potential was also tested. In Figure 8, it is seen that the solvent used for the extraction does not inhibit the growth of the tested microbial strains.

Although the inhibition zones were only observed in the case of the MOE after qualitative testing, the study continued with the quantitative determination of the antimicrobial efficiency in a liquid medium that allows for a better dispersion of the active biological compounds in the culture medium. In Table 6, it is seen that for the *E. coli* strains ATCC 13846, *E. coli* ATCC 25922 and *K. pneumoniae* ATCC 13368, the MIC value could not be determined, as microbial growth was recorded from the first well. A similar situation is highlighted in the case of *E. coli* strain ATCC 8732 in the presence of MCM-41@APTES@MOE, MCM-48@MOE and MCM-48@APTES@MOE, respectively. Among the tested strains, the most sensitive was *S. aureus* ATCC 25923, for which the average MIC values determined are in the range of 0.156–1.25 mg/mL, followed by *C. albicans* ATCC 10231 (0.078–2.5 mg/mL) and *E. faecium* ATCC 13048 (0.625–2.5mg/mL). In the case of the other strains, the degree of susceptibility to the tested samples differs according to its nature and the tested strain.

Similarly, the *E. coli* ATCC 13846; *E. coli* ATCC 25922; *E. coli* ATCC 8732; *C. albicans* ATCC 10231 and *K. pneumoniae* ATCC 13368 strains did not show susceptibility to the biologically active compounds from the MOE. In their case, the minimum inhibitory concentration was considered the undiluted extract solution (100 mg MOE of 100% concentration) for which heteroresistance was observed. In the case of the other strains tested, the average MIC value (mg) was in the range of 12.5 mg for the *E. aerogenes* ATCC 13048 strain and 41.66 mg for the *P. aeruginosa* ATCC 28792 strain (Table 7).

### 3.4. The Influence of the Mesoporous Materials on the Ability to Adhere to an Inert Substrate

Taking into account the fact that the eradication of microbial biofilms is a significant problem in clinical settings and the bacterial strains included in these structures are up to 1000 times more resistant to antibiotics compared to planktonic cells [47], an essential stage of the study involved determining the effect of the tested solutions as agents for inhibiting microbial adherence to an inert substrate. Thus, the inhibition percentage of microbial adherence was determined at sub-inhibitory MOE-loaded MSN concentrations (MIC/2 and MIC/4). The strains for which the MIC value could not be established were cultivated in the presence of 5 mg/mL/2.5 mg/mL of MOE-loaded MSNs, respectively, for 50 and 25 mg of MOE. The results varied both according to the strain tested and according to the sample with antimicrobial activity.

**Figure 9:**
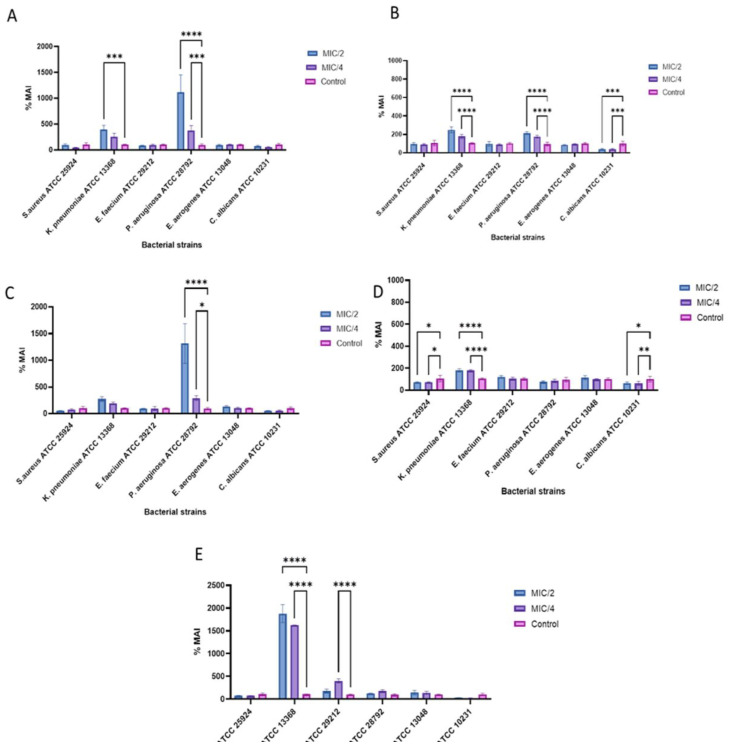
The MAI value (%) for the ATCC reference strains in the presence of MCM-41@MOE (**A**); MCM-41@APTES@MOE (**B**); MCM-48@MOE (**C**); MCM-48@APTES@MOE (**D**) and MOE (**E**) (* *p* < 0.05, ** *p* < 0.01, *** *p* < 0.001, **** *p* < 0.0001).

As seen in Figure 9A, the exposure of ATCC strains to subinhibitory concentrations of MCM-41@MOE promotes the adherence to an inert substrate of *K. pneumoniae* ATCC 13368 and *P. aeruginosa* ATCC 28792 strains, with the MAI% values at MIC/2 being 390.09% and 1115.91% and the MIC/4 values being 256.55% in the case of *K. pneumoniae* ATCC 13368 and 376.70% for *P. aeruginosa* ATCC 28792.

In contrast, in the case of the *P. aeruginosa* ATCC 28792 strain, exposure to sub-inhibitory concentrations of the plant extract does not influence its ability to adhere (Figure 9E). Thus, it can be considered that the promotion of adherence ability in the presence of MCM-41@APTES@MOE (Figure 9B) and MCM-41@MOE (Figure 9A) is a response of the strain to the stress exerted by the presence of MSNs or following particle synthesis results in a compound that promotes the adhesion of the strain to an inert substrate. Similar behavior is described for this strain in the presence of MCM-48@MOE, where the promotion of adhesion to the inert substrate was observed (MBEC%-MIC/2-1314,498 and MBEC%-MIC/4-293,144) (Figure 9C). In the presence of MCM-48@APTES@MOE (Figure 9D), although not statistically significant, a reduction in MBEC% is observed, which can confirm what was mentioned previously regarding the role of the silanizing agent in inhibiting the ability of the *P. aeruginosa* ATCC 28792 strain to adhere to the inert substrate.

In Figure 9D, except for the *K. pneumoniae* ATCC 13368 strain that was discussed previously, it is highlighted that the MCM-48@APTES@MOE sample is relevant for studies on their efficiency in eradicating microbial biofilms. In this case, the adherence capacity is inhibited for both *S. aureus* ATCC 25,924 and *C. albicans* ATCC 10231. In the case of *S. aureus* ATCC 25924, the MAI% values are 71.10% at MIC/2 and 71.75% at MIC/4, and for the *C. albicans* ATCC 10231 strain, the MAI% values are 64.77% for MIC/2 and 62.02% for MIC/4.

### 3.5. Highlighting Antimicrobial Activity against Microbial Strains Isolated from Intra-Hospital Infections

Based on the results obtained up to this point, we decided to expand the study and test the efficiency of the plant extract on *S. aureus* MRSA previously isolated from intra-hospital infections. *S. aureus* MRSA is considered one of the most versatile pathogens responsible for acquired intra-hospital infections. It is responsible for endocarditis, chronic osteomyelitis, pneumonia and osteoarthritis. Among the main mechanisms of antibiotic resistance, in the case of members of this species, we note the change in the consistency of the cell wall, the presence of a greater number of efflux pumps in the external cellular structures and biofilm formation [48,49]. In this context, highlighting a possible sensitivity to the plant extract MOE may prove to be valuable for the development of alternative strategies to combat this pathogen both in the case of systemic and local infections. In Table 8, it can be seen that the seven strains of *S. aureus* are sensitive to the antimicrobial action of the plant extract, with the MIC values being in the range of 12.5–25 mg MOE.

### 3.6. The Influence of Nanoparticle Solutions on the Ability to Secrete Soluble Virulence Factors and the Ability to Adhere to the Inert Substrate

**Figure 10:**
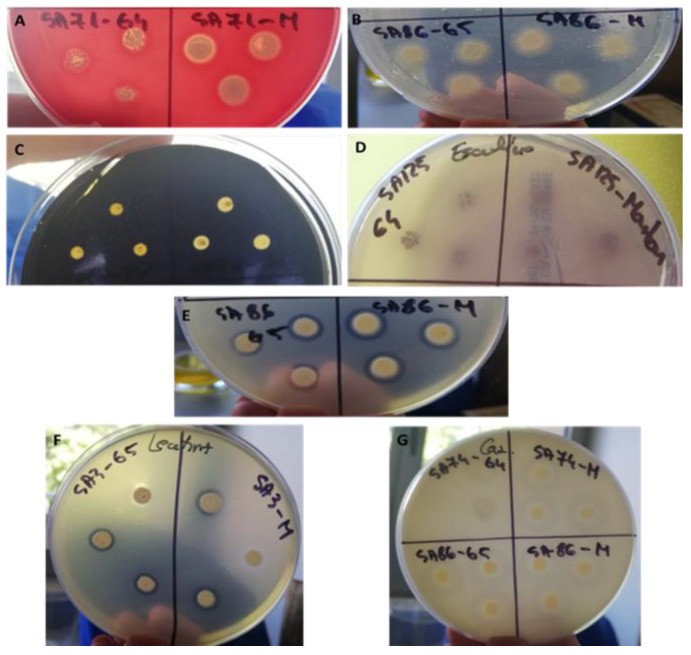
The appearance of cultures developed on specific media for the evaluation of the capacity to produce soluble virulence factors ((**A**) hemolysins; (**B**) lipases; (**C**) amylases; (**D**) esculin; (**E**) gelatinases; (**F**) lecithinases and (**G**) caseinases).

Among the *S. aureus* MRSA-tested strains, none showed the ability to adhere to the inert substrate either in the positive control or in the presence of MOE-loaded MSNs. However, they showed the ability to secrete various hydrolytic enzymes that can affect the tissues of the infected host. Thus, this study was continued by highlighting the role of the plant extract in inhibiting the production of such virulence factors. None of the strains tested showed the ability to secrete hemolysins or gelatinases. All of the strains secrete amylase, but the hydrolysis zones overlap with the diameter of the culture spots, which is why it is not possible to quantify the inhibitory effect of the plant extracts using semi-quantitative methods (Figure 10).

**Figure 11:**
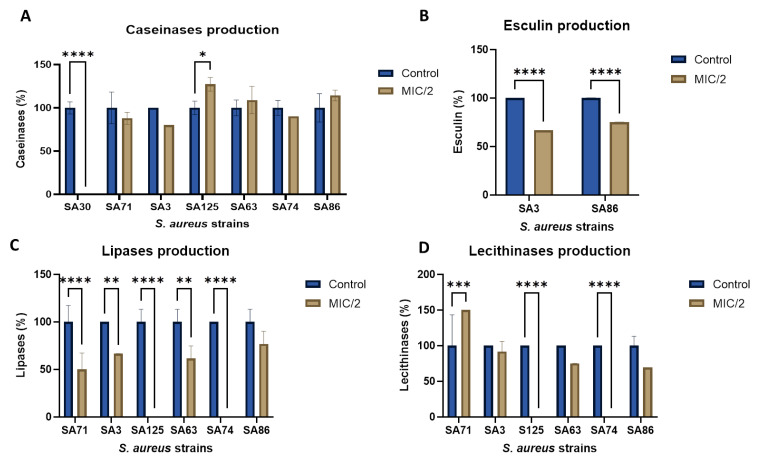
Graphical representation of percent inhibition of caseinases (**A**); esculin (**B**); lipases (**C**) and lecithinases (**D**) secretion capacity (%) for MOE plant extract tested against clinical *S. aureus* strains (* *p* < 0.05, ** *p* < 0.01, *** *p* < 0.001, **** *p* < 0.0001).

Regarding the effect on the capacity to secrete caseinases, lipases and lecithinases and the capacity to degrade esculin, the results vary depending on the tested strain and the type of factor sought. In the case of caseinases, all tested strains are positive, but it was found that the plant extract completely inhibits the capacity of the SA3 strain to secrete caseinases and has a stimulating effect in the case of the SA125 strain (inh% 127.27%) (Figure 11A).

Among the seven MRSA strains we investigated, only those encoded SA3 and SA86 are able to degrade esculin, and in the presence of sub-inhibitory concentrations of plant extract, their capacity is significantly reduced (66.66% and 75%, respectively) (Figure 11B).

In the case of lipases, six of the seven strains were able to release the fatty acid participating in the ester bond of Tween 80. For five of these, exposure to subinhibitory concentrations of the plant extract has a pronounced inhibitory effect. The capacities of the SA125 and SA74 strains are completely diminished, while for the SA71, SA3 and SA63 strains, their capacities are reduced by up to 50% (Figure 11C).

For the two strains SA125 and SA74, the inhibitory effect is also at its maximum in the case of lecithinases. In addition to these, the SA71 strain is noteworthy, in which the plant extract promoted secretion capacity (150%) (Figure 11D).

The extracts’ pharmacological properties are mostly attributed to the abundance of polyphenolic chemicals, which have antibacterial, cytotoxic and antioxidant properties. It is possible to create novel systems with biological properties for the environment and the human body by looking at the pharmacokinetics and mechanisms of action of the extracts and their active chemicals. The future potential for the creation of new systems is represented by the controlled release systems that have been developed at this point. Many materials may be functionalized to obtain controlled release systems since there are a variety of chemicals, plant extracts and essential oils available. Encapsulation carriers could be made of materials such as lipids, polymers, polysaccharides and silica. From a prospective development standpoint, *M. officinalis*’s phytochemical composition and pharmacological activities provide a chance to design novel controlled release methods that may facilitate targeted delivery [15].

One way to optimize the interaction between pharmaceuticals and mesoporous silica for more effective targeted distribution is to surface functionalize the material, as our previous study has indicated the possible controlled release of the loaded bioactive substances from mesoporous silica [32].

Beyond their ability to load pharmaceuticals of a different kind into their porous structure, MSNs have built-in advantages over other forms of nanomaterials. For example, MSNs have a higher loading capacity than solid nanoparticles like iron oxide, gold and silver nanostructures and carbon-based systems like graphene and nanotubes. MSNs outperform non-degradable gold and carbon-based structures in terms of biocompatibility. Because of their exceptional structural and chemical stability as well as their ease of functionalization, MSNs can take synthetic changes when they are loaded. However, one of silica’s most significant qualities is its extreme adaptability in creating hybrid systems that are not frequently developed with other materials. Anti-infective methods that do not require parenteral drug administration appear to be the focus of current MSN research rather than anticancer nanomedicine. For example, effective formulations based on porous silica as components for dental filling materials and cements, or even topical formulations capable of controlling bacterial infection with conventional antibiotics, have been designed [50].

Besides the use of MSNs for drug delivery with antibiotic compounds, they can be linked to compounds with antibiotic properties such as plant extracts. The effects of three essential oils, namely eucalyptus, orange and cinnamon, functionalized on MSNs were investigated by Balaure et al. [51] against the L929 mouse fibroblast cell line to assure biocompatibility as well as against clinically relevant bacterial and yeast species (*S. aureus*, *E. coli* and *C. albicans*). Chloroform, a volatile ingredient that can facilitate the loading process by reducing oil viscosity and facilitating easy evaporation, was used to load the matching essential oil into MSNs using a grinding mortar. Depending on the bacteria to be treated and the oil used, the resultant oil-loaded MSNs had notable antibacterial effects. Orange oil was efficient against *S. aureus*, whereas MSNs with eucalyptus oil had the highest potency against *C. albicans*. While cinnamon oil provided some inhibition in the *E. coli* instance, it was not nearly as active as other oil-loaded MSNs. Anyhow, at particle concentrations close to 1 µg/mL, all combinations had noteworthy benefits on the decrease in biofilm development.

A variety of studies confirmed the antibacterial properties of *M. officinalis* extract. A research group investigated the antimicrobial efficiency and chemical structures of Romanian *M. officinalis* (lemon balm) oil in 2008 and compared the in vitro antibacterial activity of lemon balm oil with that of lavender essential oil, which are commonly used in traditional medicine for their antimicrobial properties. Notable for their antibacterial properties, the most significant compounds found were trans-caryophyllene (3.57%), citronellal (3.76%) and citral (neral and geranial) (16.10%). Their qualitative antibacterial data show that Gram-positive bacteria were more susceptible to the antibacterial activity of the lemon balm oil sample than to that of the lavender oil sample; however, the *M. officinalis* oil did not affect Gram-negative bacteria. There was considerable activity against *C. albicans* in both tested oils [52]. In the same perspective, a further study showed that *M. officinalis* leaves are abundant in macro- and micromineral components, including K, Ca and Fe, among numerous others. This species’ essential oil has a high concentration of monoterpenoids, with citral making up the majority (76.780%), and varied quantities of phytochemicals. Gram-negative bacteria are more susceptible to the antibacterial properties of *M. officinalis* essential oil than Gram-positive bacteria; however, it was proven that this oil also has antifungal properties, indicating that it has potential therapeutic use. On the other hand, *M. officinalis* essential oil has weaker-than-anticipated antioxidant activity [53].

## 4. Conclusions

This study demonstrated that the natural hydroalcoholic extract of *M. officinalis* can be loaded into a mesoporous silica support and that it exhibits antimicrobial activity, especially against Gram-positive bacteria and fungal strains. The natural extract of *M. officinalis* was concentrated, obtaining a high amount of rosmarinic acid, specifically 1286 mg per 100 g of extract, determined by HPLC. In this study, four mesoporous silica systems of type MCM-41 and MCM-48 were developed, functionalized with APTES and loaded with hydroalcoholic extract of *M. officinalis*. The obtained systems have homogeneously distributed pores with particle sizes between 100 and 350 nm. The BET surface area of the systems decreased significantly after loading with MOE, which indicates a very good loading and adsorption of the extract inside the mesoporous silica pores. The obtained materials were tested against both reference strains and clinical strains belonging to Gram-positive strains that were previously isolated from intra-hospital infections (MRSA) and against Gram-negative bacteria and yeasts, observing a high sensitivity for *S. aureus* ATCC 25923 with MIC values determined in the range of 0.156–1.25 mg/mL, followed by *C. albicans* ATCC 10231 (0.078–2.5 mg/mL) and *E. faecium* ATCC 13048 (0.5 mg/mL).

Therefore, an innovative method for creating antimicrobial systems is through the integration of nanotechnology with natural extracts. Knowing how the bioactive chemicals in *M. officinalis* and the characteristics of mesoporous silica nanoparticles work together will help improve nanotechnology in the sphere of antimicrobial research. In this context, the current study provides a viable path toward the development of efficient, centered and strictly controlled antimicrobial agents with applicability across several sectors.

## Figures and Tables

**Table 1:** Types of MSNs loaded with MOE.

Material Type	Sample Code
MCM-41@*Melissa officinalis* extract	MCM-41@MOE
MCM-41@(3-aminopropyl)triethoxysilane@*Melissa officinalis* extract	MCM-41@APTES@MOE
MCM-48@*Melissa officinalis* extract	MCM-48@MOE
MCM-48@(3-aminopropyl)triethoxysilane@*Melissa officinalis* extract	MCM-48@APTES@MOE

**Table 2:** Identification and quantification of polyphenolic compounds by HPLC for *M. officinalis* extract.

Standard Substance	Estimated Quantity of Loaded Polyphenol (mg/5 mL Extract)
Chlorogenic acid	1.8
Caffeic acid	1.15
Cichoric acid	1.5
Rosmarinic acid	64.3

**Table 3:** Total polyphenolic content of *Melissa officinalis* extract.

Sample	TPC (g Caffeic Acid Equivalent/100 g Extract)	TPC (g Chlorogenic Acid Equivalent/100 g Extract)
MOE	3.14	6.33

**Table 4:** Textural parameters of MOE-loaded MSNs.

Sample	Volume of Pores cm^3^/g	BET Surface Area m^2^/g
MCM-41	0.783	1365
MCM-41@MOE	0.1024	143.9
MCM-41@APTES	0.5706	1014
MCM-41@APTES@MOE	0.0295	22.43
MCM-48	0.9423	1582
MCM-48@MOE	0.1074	143.8
MCM-48@APTES	0.7371	1555
MCM-48@APTES@MOE	0.0835	76.14

**Table 5:** Principal experimental data obtained from thermal analysis and estimated MOE load.

	Mass Loss (%) RT-125 °C	Endo (°C)	Mass Loss (%) 125–300 °C	Exo (°C)	Mass Loss (%) 300–520 °C	Exo (°C)	Estimated MOE Load (%)
MCM-41@MOE	11.89	84.1	18.52	271.2	16.00	318.0/417.3	46.45
MCM-41@APTES @MOE	4.65	90.3	20.96	272.1	18.55	321.7/421.6	35.86
MCM-48@MOE	21.70	88.4	15.39	277.4	12.82	325.9/426.6	50.02
MCM-48@APTES @MOE	10.41	84.9	15.20	276.8	16.57	323.1/432.3	32.24

**Table 6:** MIC values (mg/mL) for MCM-41@MOE, MCM-41@APTES@MOE, MCM-48@MOE and MCM-48@APTES@MOE samples.

Antimicrobial Compound		*S.aureus* ATCC 25923	*E. coli* ATCC 13846	*E. coli* ATCC 25922	*E. coli* ATCC 8732	*C. albicans* ATCC 10231	*E. faecium* ATCC 29212	*E. aerogenes* ATCC 13048	*P. aeruginosa* ATCC 28792	*K. pneumoniae* ATCC 13368
MCM-41@ MOE	MIC (mg/mL)	1.25 ± 0.0	10 ± 0.0	10 ± 0.0	0.312 ± 0.0	2.5 ± 0.0	2.5 ± 0.0	1.25 ± 0.0	10 ± 0.0	10 ± 0.0
	MIC/2 (mg/mL)	0.625	5	5	0.156	1.25	1.25	0.625	5	5
	MIC/4 (mg/mL)	0.3125	2.5	2.5	0.078	0.625	0.625	0.3125	2.5	2.5
MCM-41 @APTES @MOE	MIC (mg/mL)	0.156 ± 0.0	10 ± 0.0	10 ± 0.0	10 ± 0.0	2.5 ± 0.0	2.5 ± 0.0	5 ± 0.0	10 ± 0.0	10 ± 0.0
	MIC/2 (mg/mL)	0.078	5	5	5	1.25	1.25	2.5	5	5
	MIC/4 (mg/mL)	0.03125	2.5	2.5	2.5	0.625	0.625	1.25	2.5	2.5
MCM-48 @MOE	MIC (mg/mL)	0.625 ± 0.0	10 ± 0.0	10 ± 0.0	10 ± 0.0	2.5 ± 0.0	0.625 ± 0.0	10 ± 0.0	10 ± 0.0	10 ± 0.0
	MIC/2 (mg/mL)	0.3125	5	5	5	1.25	0.3125	5	5	5
	MIC/4 (mg/mL)	0.156	2.5	2.5	2.5	0.625	0.156	2.5	2.5	2.5
MCM-48 @APTES @MOE	MIC (mg/mL)	0.937 ± 0.54	10 ± 0.0	10 ± 0.0	10 ± 0.0	0.078 ± 0.0	2.5 ± 0.0	10 ± 0.0	1.04 ± 0.36	10 ± 0.0
	MIC/2 (mg/mL)	0.468	5	5	5	0.039	1.25	5	0.52	5
	MIC/4 (mg/mL)	0.234	2.5	2.5	2.5	0.0195	0.625	2.5	0.26	2.5

**Table 7:** MIC and corresponding MIC/2 and MIC/4 values for MOE (mg) for MAI% determination using microbial reference strains.

Strains	MIC	MIC/2	MIC/4
*S. aureus* ATCC 25923	16.66 ± 10.20	8.33	4.16
*E. coli* ATCC 13846	100 ± 0.0	50	25
*E. coli* ATCC 25922	100 ± 0.0	50	25
*E. coli* ATCC 8732	100 ± 0.0	50	25
*C. albicans* ATCC 10231	100 ± 0.0	50	25
*E. faecium* ATCC 29212	25 ± 0.0	12.5	6.25
*E. aerogenes* ATCC 13048	12.5 ± 0.0	6.25	3.12
*P. aeruginosa* ATCC 28792	41.66 ± 14.37	20.83	10.41
*K. pneumoniae* ATCC 13368	100 ± 0.0	50	25

**Table 8:** The MIC and the corresponding MIC/2 values for MOE (mg) for MAI% determination tested against clinical *S. aureus* strains.

Strains	MIC	MIC/2
*S. aureus* SA3	12.5 ± 0.0	6.25
*S. aureus* SA863	12.5 ± 0.0	6.25
*S. aureus* SA74	12.5 ± 0.0	6.25
*S. aureus* SA71	12.5 ± 0.0	6.25
*S. aureus* SA30	12.5 ± 0.0	6.25
*S. aureus* SA69	25 ± 0.0	12.5
*S. aureus* SA125	25 ± 0.0	12.5

## Data Availability

The MSN samples, MRSA isolates and data sets regarding the results are available from the authors.

## Supplementary Materials

The following supporting information can be downloaded at https://www.mdpi.com/article/10.3390/pharmaceutics16040525/s1, Figure S1: The SEM micrographs of MCM-41 are shown in (**a**,**b**), and those of MCM-48 are shown in (**c**,**d**); Figure S2: The FTIR spectra for the MCM-41, MCM-48, MCM-41@APTES and MCM-48@APTES particles and *Melissa officinalis* extract (MOE); Figure S3: The TG–DSC curves for the MCM-41, MCM-48, MCM-41@APTES and MCM-48@APTES samples.

## Abbreviations

MOE	*Melissa officinalis* extract
MCM-41	Mobile Composition of Matter No 41
MCM-48	Mobile Composition of Matter No 48
APTES	(3-aminopropyl) triethoxysilane
MCM-41@APTES	MCM-41 particles functionalized with APTES
MCM-48@APTES	MCM-48 particles functionalized with APTES
MCM-41@APTES@MOE	MCM-41@APTES particles loaded with MOE
MCM-48@APTES@MOE	MCM-48@APTES particles loaded with MOE
MSNs	mesoporous silica nanoparticles
TEOS	tetraethyl orthosilicate
CTAB	cetyltrimethylammonium bromide
TEM	transmission electron microscope
SEM	scanning electron microscopy
TG	thermogravimetric analysis
DSC	differential scanning calorimetry
FTIR	Fourier-transform infrared spectroscopy
BET	Brunauer–Emmett–Teller
HPLC	high-performance liquid chromatography
TPC	total polyphenol content
MIC	minimum inhibitory concentration
MRSA	methicillin-resistant *S. aureus*
MAI	microbial adherence inhibition
