# Supplementary material for: The Antimicrobial Potency of Mesoporous Silica Nanoparticles Loaded with Melissa officinalis Extract
Source: Pharmaceutics. 2024 Apr 10;16(4):525. doi: 10.3390/pharmaceutics16040525 (PMC11054826; doi:10.3390/pharmaceutics16040525)
Supplement: Supplementary file 1 [file pharmaceutics-16-00525-s001.zip › pharmaceutics-2892442-supplementary.pdf]

Article

The antimicrobial potency of mesoporous silica nanoparticles loaded with *Melissa officinalis* extract

Gabriela Petrişor[1,2,3,4], Ludmila Motelica[1,2,3,4], Roxana Doina Truşcă[1,2,3], Andreea-Luiza Mîrț[1,2,3,5], Gabriel Vasilievici[5], Justinian-Andrei Tomescu[6], Cristina Manea[6], Andreea Ștefania Dumbravă[7], Viorica Maria Corbu[7], Irina Gheorghe-Barbu[7], Denisa Ficai[2,3,4,8], Ovidiu-Cristian Oprea[2,3,4,8*], Bogdan-Ștefan Vasile[1,2,3,4], Anton Ficai[1,2,3,4] and Anca Daniela Raiciu[6,9]

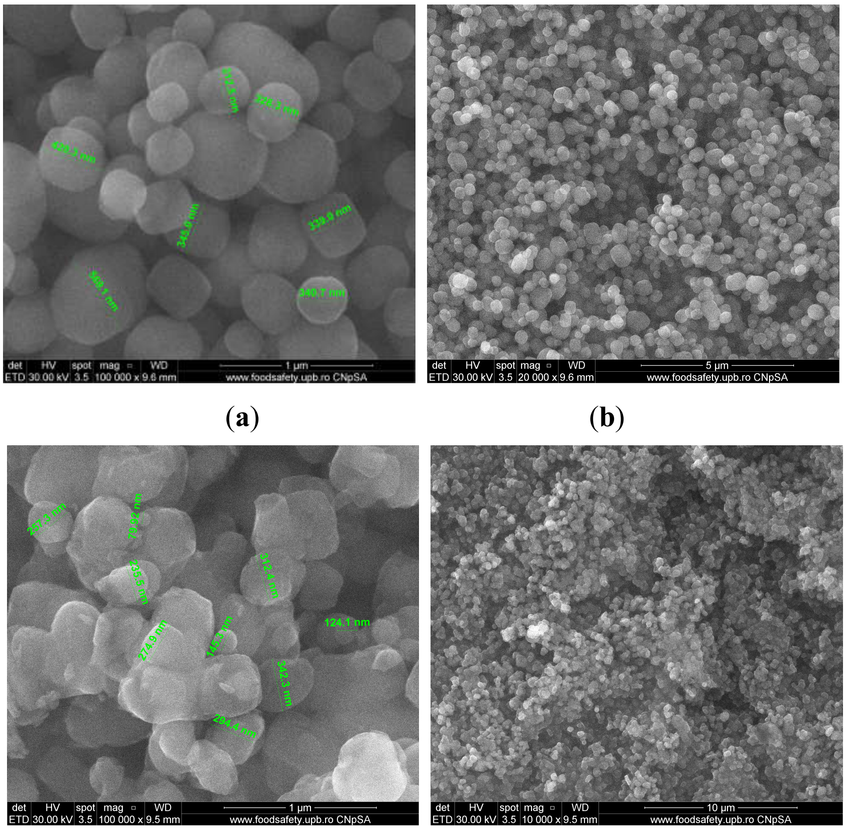

(**a**) (**b**) (**c**) (**d**)

Figure S1. The SEM micrographs of MCM-41 (**a**,**b**); MCM-48 (**c**,**d**).

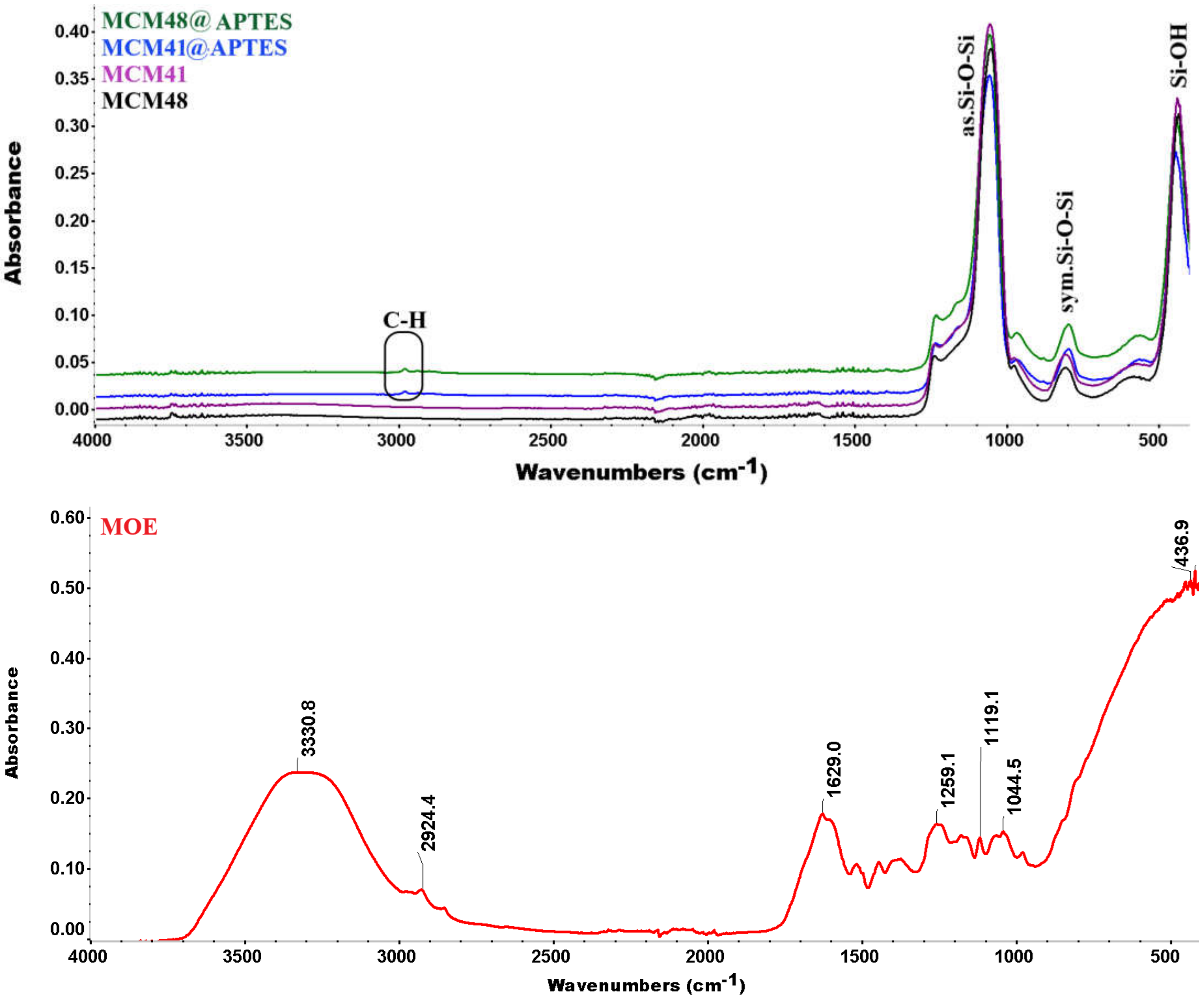

Figure S2. The FTIR spectra for the MCM-41, MCM-48, MCM-41@APTES, MCM-48@APTES particles and *Melissa officinalis* extract (MOE).

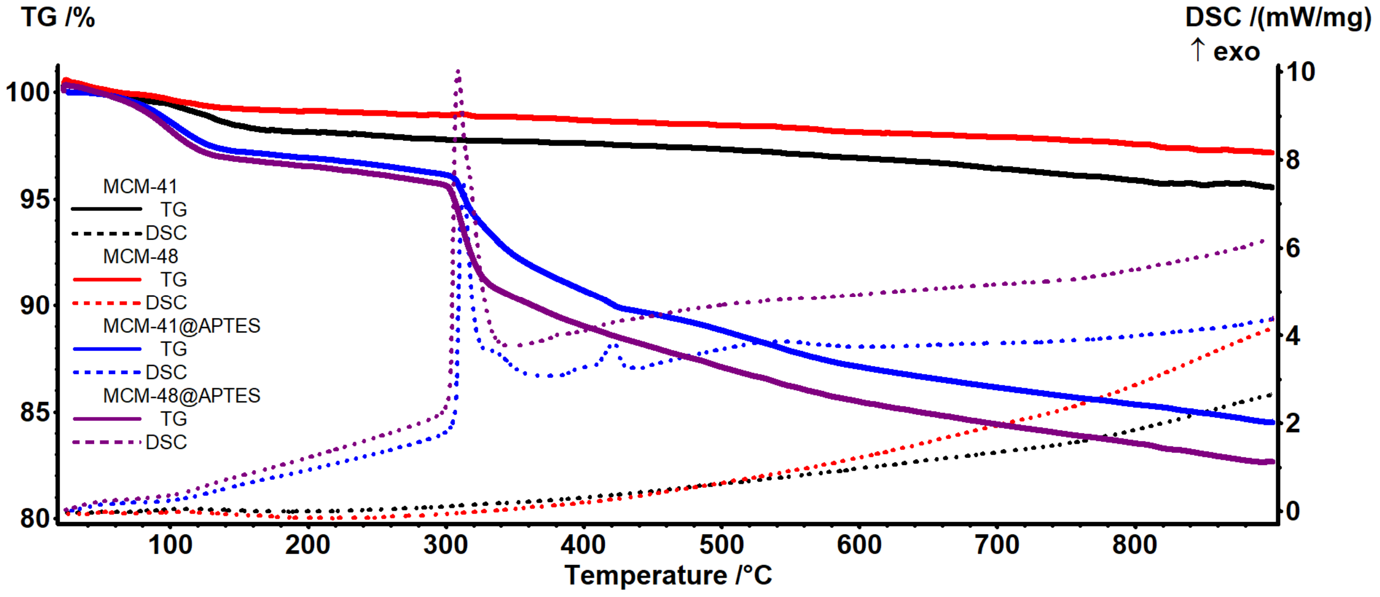

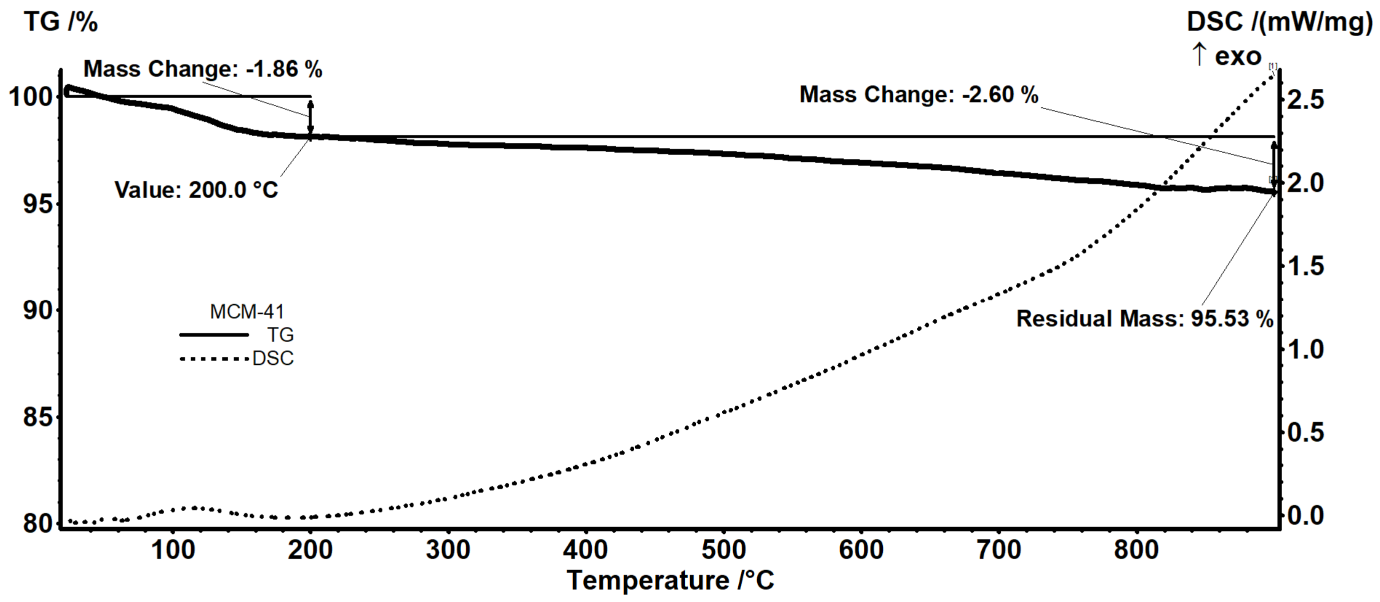
TG /%
DSC /(mW/mg)
↑ exo
Mass Change: -1.86 %
Mass Change: -2.60 %
Value: 200.0 °C
Residual Mass: 95.53 %
MCM-41
TG
DSC
Temperature /°C

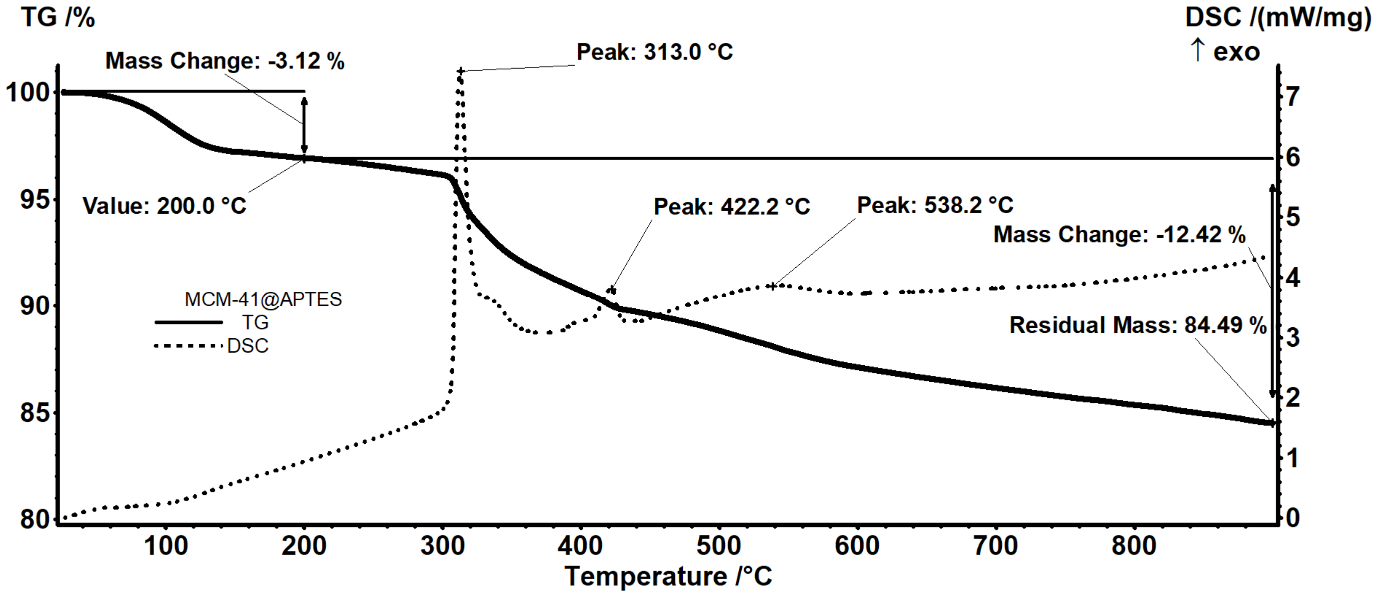
TG /%
DSC /(mW/mg)
↑ exo
Mass Change: -3.12 %
Peak: 313.0 °C
Value: 200.0 °C
Peak: 422.2 °C
Peak: 538.2 °C
Mass Change: -12.42 %
Residual Mass: 84.49 %
MCM-41@APTES
TG
DSC
Temperature /°C

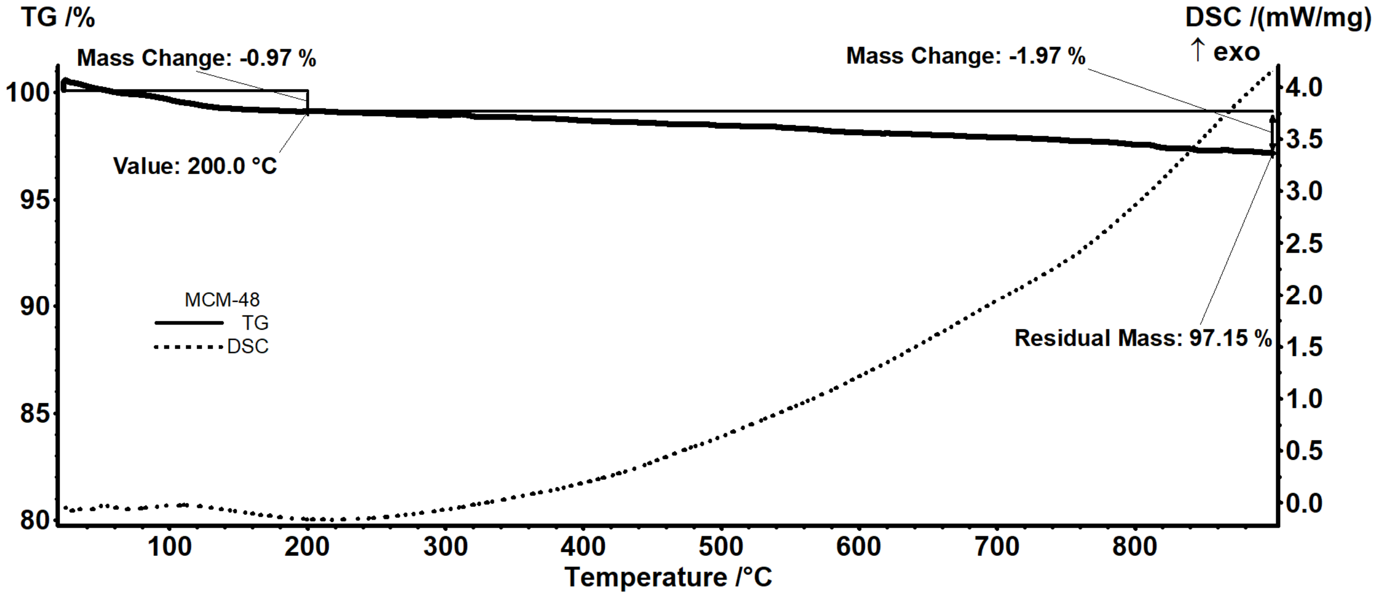
TG /%
DSC /(mW/mg)
↑ exo
Mass Change: -0.97 %
Mass Change: -1.97 %
Value: 200.0 °C
Residual Mass: 97.15 %
MCM-48
TG
DSC
Temperature /°C

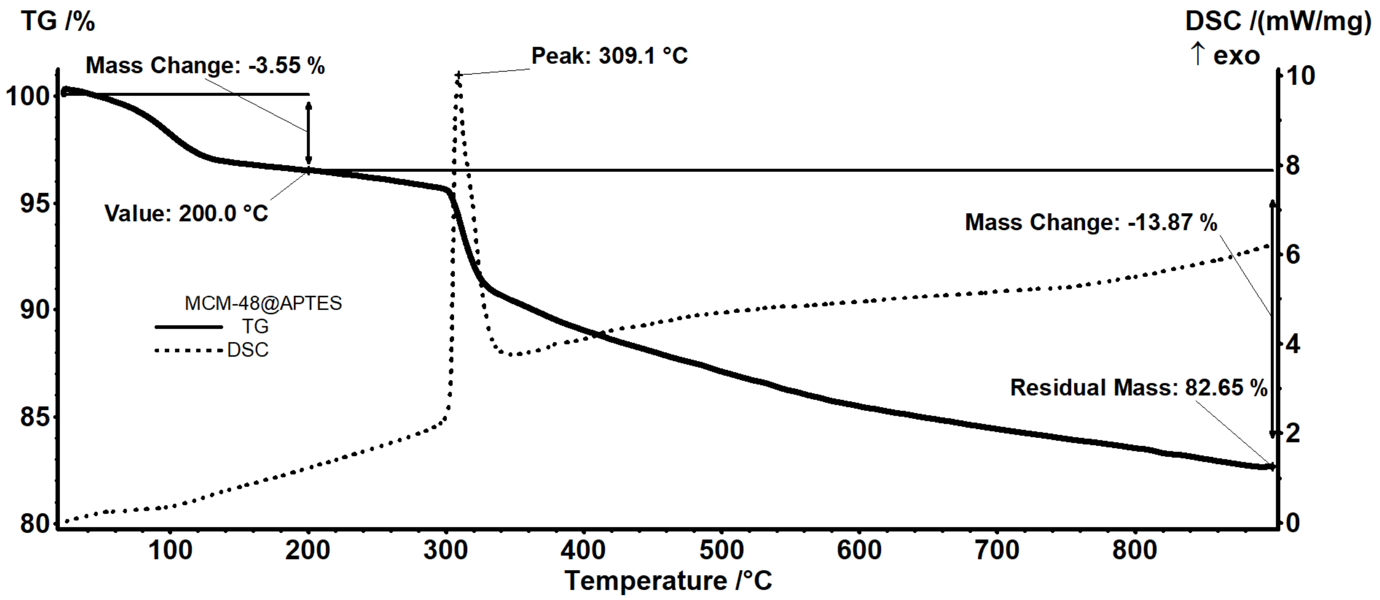

Figure S3. The TG –DSC curves for the MCM-41, MCM-48, MCM-41@APTES and MCM-48@APTES.
